# Targeting Implicit Bias in Medicine: Lessons from Art and Archaeology

**DOI:** 10.5811/westjem.2019.9.44041

**Published:** 2019-12-09

**Authors:** Amy Zeidan, Anne Tiballi, Melanie Woodward, Isha Marina Di Bartolo

**Affiliations:** * Emory University, Department of Emergency Medicine, Atlanta, Georgia; † University of Pennsylvania, Museum of Archaeology and Anthropology, Philadelphia, Pennsylvania; ‡ University of Pennsylvania, Perelman School of Medicine, Department of Internal Medicine, Philadelphia, Pennsylvania; § Rowan University School of Medicine/Cooper Hospital, Department of Internal Medicine, Camden, New Jersey

## Abstract

Implicit bias training is not currently a required component of residency education, yet implicit bias in medicine exists and may influence care provided to patients. We propose an innovative exercise that allows trainees to explore implicit bias outside of the clinical environment, in an interdisciplinary manner with museum anthropologists and archaeologists. The curriculum was designed with leaders at the Penn Museum and focuses on differentiating between objective and subjective assessments of historical objects. The first part of the exercise consists of a pre-brief, to introduce trainees to bias through the lens of an anthropologist/archaeologist. The second part guides trainees through “deep description,” where they explore objective and subjective findings of three different objects. The exercise concludes with a debrief and application of concepts learned to everyday clinical practice. This innovation was successful at introducing trainees to implicit bias in a nontraditional environment, and participants reported an improved understanding of implicit bias. Residency programs could consider partnering with local museums to implement a similar exercise as a component of conference curriculum.

## BACKGROUND

Implicit bias involves associations outside our conscious awareness that lead to misleading, often-negative evaluations of a person or patient, on the basis of characteristics such as race, ethnicity, sexual orientation, or gender. Medical professionals are not immune to implicit bias, and research has shown that the rates of implicit bias as measured by the Implicit Association Test (IAT) within the medical community are equal to that of the general population.^1^ In fact, studies have shown that physicians have a pro-white bias.^2^ These biases may influence diagnoses and ultimately, treatment decisions for patients.^3^ Leaders in healthcare are faced with the task of addressing these implicit biases by further investigating the role they play in the care of patients, and by addressing how to combat these biases. Numerous approaches have been employed; however, implicit bias education is not yet a requirement in emergency medicine (EM) residency curriculum, or more broadly, in graduate medical education curriculum.^4^,^5^,^6^ We provide a description of an educational approach using archaeologic concepts to introduce implicit bias to trainees.

## OBJECTIVES

There are many challenges in developing implicit bias training during residency. Because of the limited time available to residents in their training, educators must try to coalesce as much content as possible into a limited amount of time. Furthermore, implicit bias training has been frequently associated with increasing rates of anxiety and disengagement.^5^ We postulated that decoupling the training from the hospital and direct clinical experiences and having the discussions in a low-stakes environment, such as a museum, could be an effective way to introduce the concept of implicit bias to trainees. Therefore, the educational objective of this curriculum was to develop an exercise that can be performed during residency conference that allows the trainee to explore implicit bias through the lens of an archaeologist, using objects and artwork, rather than clinical settings.

## CURRICULAR DESIGN

This curriculum was designed with anthropology and archaeology experts at the Penn Museum of Archaeology and Anthropology. Similar collaborations have been implemented between Yale Medical School and the Center for British Art and Harvard Medical School and the Museum of Fine Arts Boston.^7^ The exercise consists of three components: a pre-brief with session leaders; viewing three objects using a tool called “deep description;” and a post-session reflective discussion.

During the pre-brief, trainees are introduced to the kinds of biases that exist while creating narratives and explanations for the ancient past by museum anthropologists and archaeologists. The group leader emphasizes that archaeologists work with an incomplete record, and must “fill in the gaps” in the histories they write, but that this process necessarily introduces the scholar’s own bias into the story of the past.

After this introduction, trainees visit three objects and are asked to practice performing “deep description.” Deep description is the process of analyzing an object with the intent of understanding human behavior and activities. It pushes the observer to think critically about each small detail of an object with the purpose of understanding the intent of the creator, and how the object represents traditions, cultures, and communities. Importantly, this technique encourages the participant to separate objective findings from subjective evaluations.

**Figure 1:**
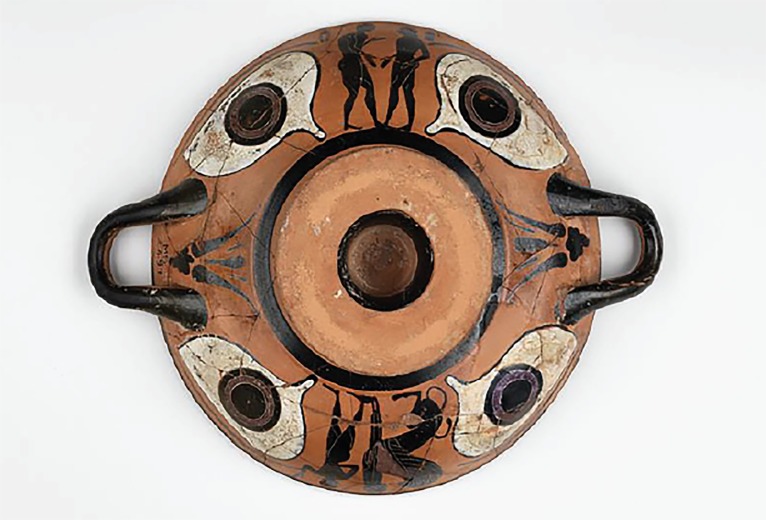
Sample object with aesthetic and anthropologic prompts.

Each object session begins with five minutes of silent observation and proceeds to observations and inferences based on those observations. Prompts included both aesthetic questions that required the trainees to closely examine the objects, and anthropological questions that encouraged them to make inferences about how the object was made and used and the people who made and used it (Figure 1).

Using deep description in this setting allows trainees to distinguish objective findings, “the handle is broken,” from subjective evaluations, “the bowl was thrown away after it broke, since it could no longer be used.” Interestingly, further examination might show that the bowl was repaired in antiquity and continued to be used, suggesting that the owner valued it highly or could not afford to replace it. Exploring objective and subjective findings assists trainees in acknowledging that biases exist and are almost inevitable.

Finally, trainees participated in a reflective discussion to share their experiences of using deep description and how these experiences allowed the observer to understand implicit bias outside of a clinical environment. Participants easily moved between the specific, archaeological aspects of the curriculum and their experiences in clinical settings. Assumptions about objects had direct parallels in assumptions about patients, and participants were aware of the ways in which the need to create a complete “story” can often be influenced by their own biases.

## IMPACT/EFFECTIVENESS

Participants were asked voluntarily and anonymously to complete a survey prior to the start of the exercise. Participants then completed the same survey at the conclusion of the exercise after the reflective discussion. The pre and post survey included six questions that aimed to assess the participant’s understanding of implicit bias including questions such as, “I believe that addressing implicit bias can improve patient care.” The survey also included short-answer questions about prior experiences with implicit bias. Results were collected and collated by the authors, and analyzed using Excel (Microsoft Corp., Redmond, WA) statistics.

**Table:** 

Aesthetic	Anthropological
What colors do you see? How are they combined?	Who made this object?
What is the object made out of? Why did the maker use that material?	Who used it?
What patterns do you see? How do they repeat?	How old is it?
Do you see any artisanal “mistakes”?	Was the user wealthy? Poor? Male? Female?
What words would you use to describe the object?	What does this object do?
What do you feel when you look at this object?	What meaning or idea does it convey?

A total of 26 participants completed this workshop as a compulsory part of their curriculum. There were three workshops in total, two for internal medicine (IM) residents and one for EM residents/clerkship students. All seven IM residents completed the pre/post survey. There were 19 total participants in the EM group consisting of EM residents and medical students participating in their EM clerkship. In the EM group, 11 participants completed both the pre/post survey (four medical students, seven EM residents). Most participants had minimal to no training on implicit bias prior to this exercise (89%). Participants reported having a better understanding of implicit bias after the exercise (67%) and reporting feeling more empowered to address their biases after completion of the exercise (61%). Most participants reported learning something new or surprising from the session (78%), specifically commenting on how “quickly we jump to conclusions and assumptions” and how challenging it is to “separate observation from interpretation.”

Finally, participants expressed wanting more time for this and similar sessions, specifically recommending a longer “lecture” on implicit bias, longitudinal exercises, and a more thorough discussion on interventions that may help prevent implicit bias from affecting clinical care. In the future, we recommend expanding the allotted time of the session to at least four hours, or dividing the session into two components over a two-week period to provide ample time for reflective discussion.

This workshop was effective at delivering content on the subject of implicit bias, and it fostered conversations about bias in a low-stakes, interdisciplinary environment. It should be noted that this exercise has not been shown to alter an individual’s implicit biases, or evaluate how this may affect clinical care and outcomes, which is an important area for further investigation.
